# A New Theory of the Formation of Coal

**Published:** 1882-06

**Authors:** 


### A NEW THEORY OF THE FORMATION OF COAL.

After a protracted microscopic study of coal, Prof. Reinsch has come to the conclusion that coal was not derived from land plants, but chiefly from microscopic forms of "a lower order of protoplasm." He holds that plants of a higher order have contributed but a fraction of the mass of coal veins, however, numerous they may have been in some instances. In a recent lecture, stating his conclusions, Prof. Reinsch referred to the fact that Dr. Muck, of Bochum, held that algæ have mainly contributed to the formation of coal, and that marine plants were rarely found in coal because of their tendency to decompose, and that calcareous remains of mollusks disappeared on account of the rapid formation of carbonic acid during the process of carbonization.

